# Correlation between Tomography Scan Findings and Clinical Presentation and Treatment Outcomes in Patients with Orbital Floor Fractures

**DOI:** 10.3390/diagnostics14030245

**Published:** 2024-01-24

**Authors:** Łukasz Stopa, Wojciech Stopa, Zygmunt Stopa

**Affiliations:** 1 Department of Descriptive and Clinical Anatomy, Center for Biostructure Research, Medical University of Warsaw, 02-091 Warsaw, Poland; 2 Department of Craniomaxillofacial Surgery, Oral Surgery and Implantology, Medical University of Warsaw, 02-091 Warsaw, Poland

**Keywords:** orbital fracture, computed tomography, craniofacial trauma, reconstructive surgery, symptoms

## Abstract

*Background*: Orbital floor fractures involve damage to the orbital floor but not the infraorbital margin. Despite intensive research, they remain a controversial topic. The aim of this study was to investigate the relationship between parameters gathered by means of computed tomography (CT), the clinical presentation, and treatment outcomes, in patients suffering from orbital floor fractures. *Methods*: Forty patients with orbital floor fractures were included in this study. Information regarding diplopia, impaired ocular mobility, asymmetric eyeball placement, and infraorbital paresis was gathered from the medical records. Nine CT-based parameters were assessed. Two parameters were calculated, based on them. The follow-up data of 30 patients were analyzed. The results were statistically evaluated. The significance level was *p* < 0.05. *Results*: Statistical evaluation revealed multiple correlations between CT-based findings, symptoms, and treatment results. Among others, the hernia into the maxillary sinus was significantly larger in patients without improvement in infraorbital paresis (*p* = 0.0031) and without improvement in assymetric eyeball placement (*p* = 0.0037). There was no correlation between the entrapment of the rectus inferior muscle and impaired ocular mobility (*p* = 0.664431; *p* = 0.420289) and between the direct fracture of the infraorbital canal and infraorbital paresis (*p* = 0.371102). *Conclusions*: The widely assumed thesis that impaired ocular mobility in orbital fractures is caused by entrapment of the rectus inferior muscle is disproved by CT-based data. CT-based findings, symptoms, and treatment results in patients with orbital floor fractures were significantly correlated. A large hernia may be a negative prognostic factor.

## 1. Introduction

Orbital floor fractures have remained a source of enduring controversy and intrigue within the field of maxillofacial surgery, eliciting ongoing discussions and scholarly investigations. According to the classification system developed by Professor Hubert Wanyura, widely adopted in Poland, these fractures fall within the category of low facial injuries sustained due to lateral force application [1]. They bear a distinctive hallmark: the presence of an orbital floor fracture, unaccompanied by a fracture of the infraorbital margin [1]. It is important to note that this classification system also encompasses fractures of the medial wall. However, a contrasting perspective emerges when considering the AO (Arbeitsgemeinschaft für Osteosynthesefragen) Craniomaxillofacial Classification, which firmly distinguishes between orbital floor and medial wall fractures [2]. While the term “blowout” fractures is often casually assigned to orbital floor fractures [3], this designation itself evokes debate, as it refers to the controversial pathomechanisms governing these injuries. Some scholars posit that such fractures result from a sudden surge in intraorbital pressure subsequent to an direct impact on the eyeball, ultimately culminating in the fracturing of the most vulnerable wall of the orbit: the orbital floor itself [1,4,5,6,7,8]. Conversely, an opposing viewpoint has been articulated, suggesting that such injuries are infrequent, with the majority of impacts predominantly involving the infraorbital margin. In such scenarios, the fracture is believed to stem from the direct transmission of force to the orbital floor [4,5,6,7,8,9]. Over the years, numerous studies have been conducted, yet clear-cut guidelines for the treatment of orbital floor fractures have yet to emerge [10]. It is generally surmised that surgical intervention is warranted in cases marked by enophthalmos and persistent diplopia, with the presence or absence of infraorbital paresis emerging as an additional factor influencing the treatment decision-making process [3]. In terms of surgical interventions, the reconstruction of the orbital floor can be accomplished through a variety of materials, ranging from the employment of titanium to the utilization of bioresorbable substances and autogenous bone grafts. Nevertheless, the precise criteria governing the selection of a particular treatment modality and the potential superiority of certain surgical techniques over others remain fervently debated topics within the annals of medical literature [10,11,12,13]. The central objective of this study was to investigate the relationship between the parameters gathered through the use of computed tomography (CT) scans and the clinical presentation, as well as the outcomes of treatment, in patients suffering from orbital floor fractures.

## 2. Materials and Methods

This retrospective study included patients treated for orbital floor fractures in the clinic between January 2017 and October 2019 for whom computed tomography (CT) scans of sufficient quality were available. The exclusion criteria were the presence of additional facial fractures or pathologies and incomplete or unclear medical records. Therefore, 40 patients were included in this study. The study was conducted in compliance with the legal and ethical standards applicable in Poland and the European Union.

**Figure 1:**
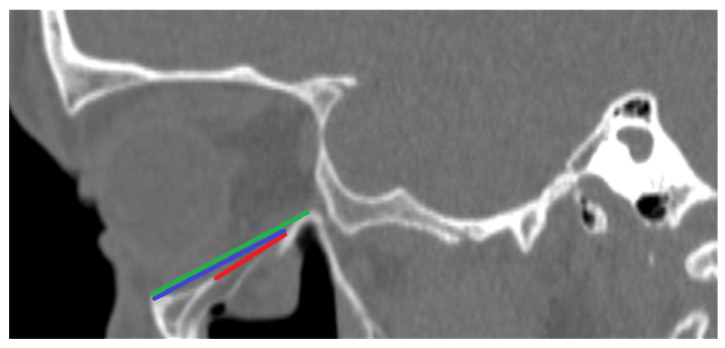
CT-scan. Sagittal view of a fractured orbit. Parameter I marked red, Parameter II marked blue, Parameter III marked green.

**Figure 2:**
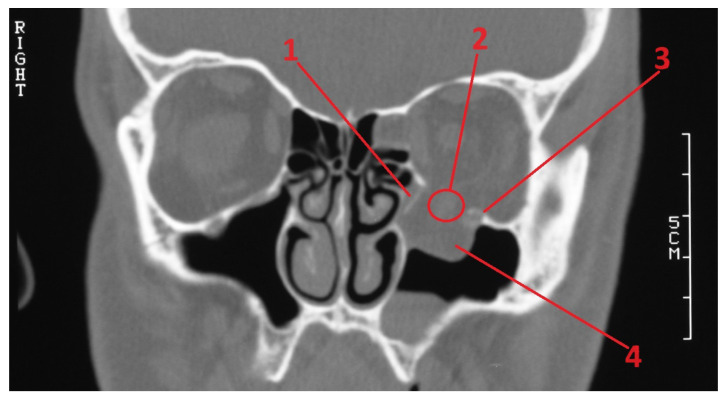
CT-scan. Coronal view of a fractured orbit on the left side of the patient. Following elements marked: 1—bony fragment; 2—displaced rectus inferior muscle; 3—infraorbital canal; 4—hernia in the maxillary sinus.

**Figure 3:**
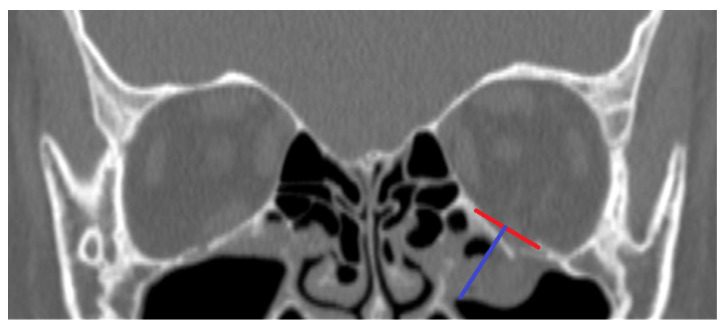
CT scan. Coronal view of a fractured orbit on the left side of the patient. Parameter VI marked red, Parameter VII marked blue.

By using the patients’ medical records, information regarding the presence of diplopia, impaired ocular mobility, asymmetric eyeball placement, and infraorbital paresis before treatment was collected. A symptom was considered “present” if it occurred at least once from admission to the hospital until the moment surgical treatment was performed, or until the patient was discharged who had received conservative treatment. The follow-up of patients for whom data were available (30 patients in total—the remaining 10 patients did not show up at follow-up visits) was also evaluated. Information regarding the aforementioned symptoms was collected. (For details, see Appendix A). By using the Weasis DICOM viewer (v3.5.4, Hewlett Packard, Palo Alto, CA, USA), nine computed tomography-based parameters were evaluated:

- PI—Largest length of the fracture in the sagittal plane in millimeters (See Figure 1);
- PII—Distance between the end of the fracture and the infraorbital margin in the sagittal plane in millimeters (See Figure 1);
- PIII—Distance between the infraorbital margin and the end of the orbit in the sagittal plane in millimeters (See Figure 1);
- PIV—Whether the fracture includes the medial wall of the orbit; (See Figure 2);
- PV—Relation between the fracture and the infraorbital nerve [3 possibilities: (A) the fracture does not include the infraorbital canal and (B) the fracture goes directly through the infraorbital canal, and (C) the infraorbital canal is within a fractured bone fragment] (See Figure 2);
- PVI—Largest width of the fracture in the coronal plane in millimeters (See Figure 3);
- PVII—Size of a hernia into the maxillary sinus in the coronal plane in millimeters (See Figure 3);
- PVIII—Position of the fractured bone fragments (i.e., presence or absence of a trap-door formation) (See Figure 2); and
- PIX—Displacement of the rectus inferior muscle [3 possibilities: (A) muscle not displaced, (B) muscle displaced to the level of the orbital floor, and (C) muscle displaced into the maxillary sinus] (See Figure 2).

The approximate surface of the fracture (mm^2^; Parameter X–PX), the product of the largest length and width of the fracture divided by 2, and the distance between the end of the fracture and the end of the orbit in millimeters (Parameter XI–PXI) were calculated. The results were statistically evaluated. The significance level was set at *p* < 0.05. For details on the methodology of statistical evaluation, see Appendix B.

## 3. Results

A total of 8 patients included in the study were female, while 32 were male. For the patients for whom follow-up data were available, this was 6 and 24, respectively. The mean age of the patients was 39 years (32 for females and 41 for males). The data regarding the cause of the injury can be found in Table 1.

At admission, 24 fractures were located on the left side, with 16 located on the right. In terms of the treatment method, 19 patients were treated conservatively, and 21 by means of surgical reconstruction of the orbital floor. In 14 cases, resorbable material had been used, with titanium being used in the remaining 7 cases. An autologous bone graft had not been used in any of the patients.

In terms of follow-up data, 17 fractures were located on the left side, and 13 on the right. A total of 12 of the patients in the follow-up had been treated conservatively, while the remaining 18 were treated surgically. In terms of the material used for the reconstruction of the orbital floor, resorbable material had been used 12 times and titanium 6 times.

A total of 26 patients reported experiencing diplopia prior to the commencement of their treatment, whereas 8 patients reported experiencing infraorbital paresis on the affected side. Additionally, among the patients, 19 individuals exhibited impaired ocular mobility, while 9 displayed asymmetric eyeball placement. It is important to note that due to various factors such as the severity of the injury or alcohol intoxication at admission, data concerning the presence of diplopia, impaired ocular mobility, and asymmetric eyeball placement prior to treatment initiation were unavailable for 2, 6, and 5 patients, respectively. Data regarding infraorbital paresis were available in all cases. For further details on this topic, see Appendix C.

Regarding follow-up data, a total of nine patients reported the persistence of diplopia after the completion of their treatment, with six cases following surgical intervention and three cases after conservative treatment. Moreover, four patients reported experiencing infraorbital paresis post-treatment, with all four cases occurring following surgical treatment. In contrast, none of the patients reported infraorbital paresis after conservative treatment. Furthermore, impaired ocular mobility persisted in two patients, and only one patient exhibited continued asymmetric eyeball placement following treatment.

Additionally, an assessment was conducted to determine whether diplopia had subsided after treatment. This evaluation was motivated by the possibility of diplopia persisting as a complication of surgical treatment, even in patients who had not reported it upon admission. It was observed that diplopia had resolved in a total of 11 patients, with 8 cases resolving after surgical treatment and 3 cases resolving after conservative treatment. On the other hand, diplopia did not subside in eight patients, with all eight cases occurring following surgical treatment and none after conservative treatment.

The results of relevant parameter measurements in relation to diplopia as well as the *p*-values of the appropriate statistical tests (see Appendix B) are presented in Table 2. The average surface of the fracture was 166 mm^2^ for patients reporting diplopia before treatment and 205 mm^2^ for patients who did not report diplopia before treatment, while the results were 163 mm^2^ for patients with diplopia after treatment and 197 mm^2^ for patients without diplopia after treatment. The mean values for patients in whom diplopia subsided after treatment and for patients without improvement in terms of diplopia after treatment were 214 mm^2^ and 178 mm^2^, respectively. (See Appendix D for details regarding how patients were classified regarding improvement).

The results of the measurements of the parameters (and relevant *p*-values; for details, see Appendix B) in relation to asymmetrical eyeball placement and impaired eyeball mobility and in relation to infraorbital paresis are presented in Table 3 and Table 4, respectively.

The statistical evaluation proved that patients with asymmetric eyeball placement before treatment had significantly longer orbits (*p* = 0.0146), significantly longer fractures in the sagittal plane (*p* = 0.0225), and significantly larger fracture surfaces (*p* = 0.0439). Moreover, a statistically significant correlation exists between the presence of asymmetric eyeball placement before treatment and the presence of a medial wall fracture (*p* = 0.00342).

Furthermore, patients with improvement in terms of asymmetric eyeball placement (*p* = 0.0037) and in terms of infraorbital paresis (*p* = 0.0031) had significantly smaller hernias into the maxillary sinus. Additionally, there was a correlation between the presence of a medial wall fracture and improvement in terms of asymmetric eyeball placement (*p* = 0.0473).

For the other symptom/parameter combinations, no statistically significant results were found.

Further significant results have been observed in terms of the age and sex of the patients as well as the side of the fracture.

To begin with, patients with a fracture located on the left side significantly more often displayed asymmetric eyeball placement (*p* = 0.0131), while if the fracture was located on the right side, the hospitalization times had been significantly longer (*p* = 0.0453). Moreover, patients presenting diplopia before treatment (*p* = 0.0334) as well as after treatment (*p* = 0.0491) were significantly younger. Additionally, there was a correlation between female sex and the presence of infraorbital paresis after treatment (*p* = 0.0181).

A controversy was observed in terms of the results regarding the correlation between impaired ocular mobility and parameter V. The result of the Chi-squared test was significant (*p* = 0.0479), while the result of Fisher’s exact test was not, although barely so (*p* = 0.0534).

## 4. Discussion

The findings of this study contribute to the ongoing and fierce discourse that revolves around the intricate biomechanics of orbital floor fractures. Over the years, numerous studies have probed this complex topic, especially in terms of how the fracture comes into being. Some authors opt for the “blowout” mechanism; others suggest the force transfer through the infraorbiital margin [5,6,7,8]. Paradoxically, despite this substantial body of research, the pathophysiology behind the diverse spectrum of symptoms that manifest in the context of orbital fractures remains a comparatively underexplored field within the scientific landscape. This absence of comprehensive studies could be attributed to the superficially apparent origins of these symptoms, thereby leading to a preconceived notion that further investigation may be superfluous. However, the results yielded from the statistical analysis performed in this study serve to challenge certain established yet empirically unsubstantiated assumptions.

**Figure 4:**
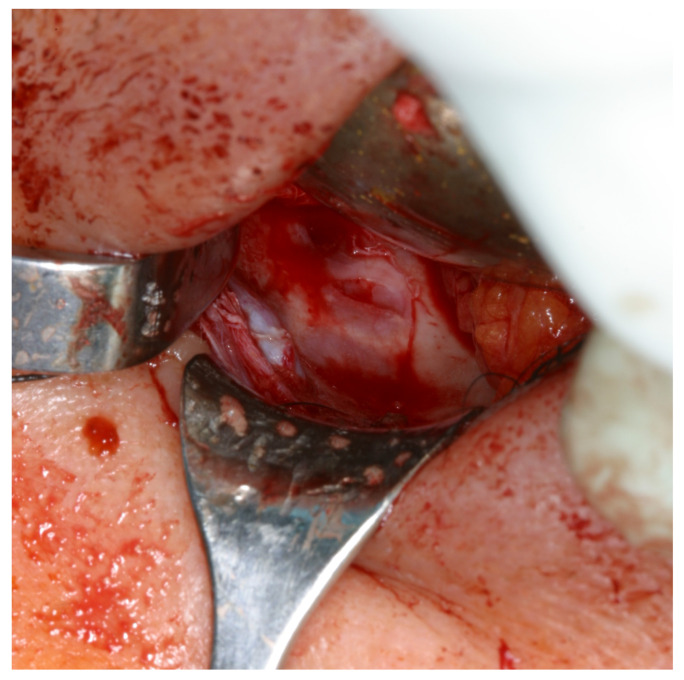
Transconjunctival approach to the right orbital floor. A narrow fracture line visible.

To begin with, it stands to reason that the presence of a medial wall fracture demonstrates a statistically significant correlation with the presence of asymmetrical eyeball placement. The structural disruption of the medial wall probably allows for the medial movement of the eyeball, subsequently resulting in observable ocular asymmetry. The same logic can be applied to the larger fracture surfaces and larger lengths of the fractures in patients with asymmetric eyeball placement. This connections, though seemingly straightforward, curiously do not extend to other symptoms of orbital fractures—diplopia and impaired ocular mobility—as no statistically significant correlation between the aforementioned symptoms and the presence of a medial wall fracture, the fracture surface, or length of the fracture has been found. More intricately even, no statistical correlation was observed between the presence of diplopia, impaired ocular mobility, and asymmetric eyeball placement. This in itself is at least intriguing, as common sense would suggest that diplopia is the result of the two aforementioned pathologies. Furthermore, in most facial fractures, an assumption is that larger fractures have a worse prognosis and are harder to treat [14,15]. The contrasting situation in orbital floor fractures may be explained by the difficult nature of the surgical procedure. Due to narrow access and a limited field of view, larger fractures are possibly easier to reconstruct, which would thus compensate for their more significant clinical implications. (See Figure 4) Sadly, the data gathered in this study do not allow us to further explore this topic. Yet, the results demonstrate that further research is warranted.

At the same time, other findings may evoke even more surprise. There were no significant results regarding the size and surface of the fracture and the presence of infraorbital paresis. Moreover, the absence of a significant correlation between the inclusion of the infraorbital canal within the fracture, as visualized through the CT scans, and the incidence of infraorbital paresis defies a simplistic explanation, rendering this an enigma. One could argue that the sample size could have been too small. At the same time, it seems more likely that the results show that the mechanism by which the infraorbital nerve is damaged due to infraorbital fractures is more complex than it might seem at first. A possible explanation, alternative to widely assumed direct damage due to a fractured infraorbital canal, would be ischemia, or possibly pressure on the nerve by damaged tissues [16]. Another possibility is subtle damage to the thin orbital floor in the region of the infraorbital canal, to a degree too minuscule to be registered by means of a standard CT scan.

**Figure 5:**
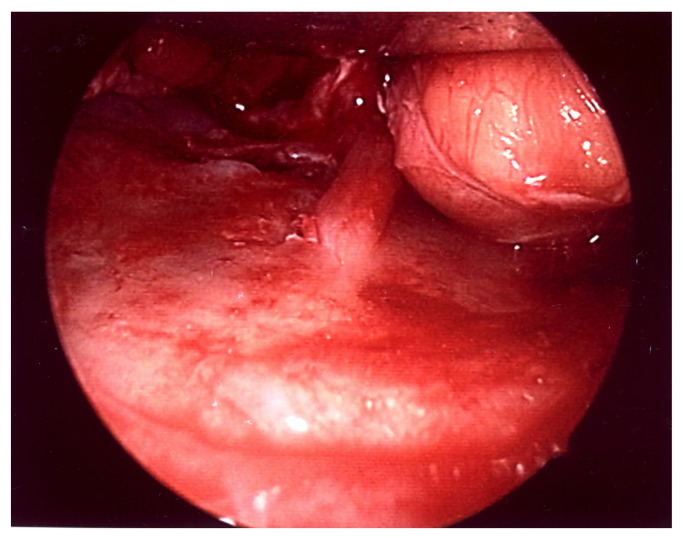
Endoscopic view of the left orbital floor reached by the transconjunctival approach. A wide fracture visible.

Another very important point are the results regarding the size of the hernia into the maxillary sinus. The lack of significant correlations before treatment and significant results in relation to improvement after treatment suggest that a large size of the hernia could potentially be a negative prognostic factor. These findings are in a sense consistent with the current literature, as some authors have suggested the volume of the hernia as a potential indication for surgical treatment [17]. This is obviously not the same conclusion, as it is related to the choice of the treatment method and not directly to the prognosis; however, in both cases, there is a suggestion that the size of the hernia is clinically significant. (See Figure 5).

At the same time, it is important to note that no significant correlation has been observed between the distance to the end of the orbit and the symptoms. This would suggest that the proximity of the fracture to the common tendinous ring, which is the origin of the four recti muscles [18], does not increase the risk of damage to those structures.

As mentioned in the section “Results”, the two statistical tests were not unanimous in terms of the correlation between the involvement of the infaorbital canal in the fracture and impaired ocular mobility, with the Chi-squared test suggesting significance and Fisher’s test giving results slightly above the significance level. This could easily be dismissed as coincidental, resulting from too small a sample. Together with the significantly larger length of the orbit in patients with asymmetric eyeball placement, this could, however, lead to the conclusion that the biomechanical attributes of the orbit, as well as the pathophysiology of symptoms manifested in the context of orbital floor fractures, are considerably more nuanced and complex than heretofore posited.

In terms of other significant results (e.g., the correlations between female sex and infraorbital paresis as well as between younger age and diplopia), it is, in most cases, hard to present logical, coherent, and evidence-based explanations. Our study does not include data needed to further dwell into those topics. The potential hypotheses would be thus based on personal bias rather than merit.

However, the highlight of this study is the complete absence of any significant results regarding the displacement of the rectus inferior muscle or the formation of a “trap door” by broken bony fragments. This finding is in stark contrast to the popular belief, widely assumed, that ocular mobility in patients with orbital fractures becomes impaired because of the displacement and entrapment of the rectus inferior muscle [3,19,20]. Notably, however, these beliefs, though widely held, are grounded more in common sense and anecdotal observation than on a foundation of empirical evidence derived from substantial research, as we have not found any comprehensive research paper that could prove them. In this context, the results of this study demonstrate that the mechanism of impaired ocular mobility in orbital floor fractures is different to what is widely claimed. Moreover, increased thickness of the rectus inferior muscle has been observed on the affected side in patients with orbital floor fractures [21]. When viewed in conjunction with our findings, this particular observation hints that the impaired ocular mobility in patients suffering from orbital floor fracture could potentially be a consequence of direct injury to the rectus inferior muscle or ischemia triggered by the swelling of surrounding tissues, rather than physical entrapment of the muscle itself.

## 5. Conclusions

The biomechanisms commonly assumed to cause orbital floor fracture symptoms are disproved by CT-based data. A significant correlation existed among CT-based findings, symptoms, and treatment results in patients with orbital floor fractures. A small fracture surface area may be a negative prognostic factor.

## Figures and Tables

**Table 1:** Cause of the injury.

Sex	Battery	Sport Injury	Traffic Accident	Fall	Other/Unknown/No Data
Male	65.6%	9.4%	0 %	15.6%	9.4%
Female	37.5%	12.5%	12.5%	25 %	12.5%
Total	60%	10 %	2.5%	17.5%	10%

**Table 2:** Results regarding diplopia before treatment (BT), after any kind of treatment (AT), and improvement of diplopia after treatment (IAT). The results for diplopia present are presented above the results for diplopia absent. The results for PIV and PVIII are presented as YES/NO and for PIX as A/B/C. The *p*-values for the appropriate statistical tests are presented below.

	PI	PII	PIII	PIV	PVI	PVII	PVIII	PIX	PXI
BT	21.7 mm	29.7 mm	32.8 mm	8/18	16.1 mm	9.4 mm	19/7	11/11/4	3.2 mm
	24 mm	29.9 mm	33.7 mm	6/5	16.9 mm	10.4 mm	6/5	3/6/2	3.9 mm
	0.2525	0.8444	0.4090	0.1729	0.6492	0.2529	0.2711	0.6869	0.4277
AT	19.8 mm	28.7 mm	32.2 mm	4/5	15.8 mm	10.3 mm	8/1	3/4/2	3.5 mm
	23.4 mm	29.6 mm	33.2 mm	7/13	16.1 mm	9.6 mm	12/8	9/9/2	3.6 mm
	0.0978	0.4992	0.4337	0.6277	0.8575	0.5691	0.1198	0.6451	0.9763
IAT	23.4 mm	30.5 mm	32.9 mm	3/7	17.0 mm	10.1 mm	6/4	5/4/1	2.4 mm
	22 mm	29.4 mm	32.9 mm	8/10	15.8 mm	10.0 mm	13/5	6/9/3	3.6 mm
	0.5232	0.2664	0.9736	0.4533	0.4688	0.9288	0.5070	0.6727	0.2084

**Table 3:** Results regarding asymmetric eyeball placement (AEP) and impaired ocular mobility (IOM) before treatment. The results for the symptom present are presented above the results for the symptom absent. The results for PIV and PVIII are presented as YES/NO and for PIX as A/B/C. The *p*-values for the appropriate statistical tests are presented below.

	PI	PII	PIII	PIV	PVI	PVII	PVIII	PIX	PX	PXI
AEP	26.2 mm	30.4 mm	35.0 mm	7/2	18.1 mm	8.3 mm	5/4	3/3/3	242 mm^2^	4.6 mm
	21.3 mm	29.0 mm	32.3 mm	6/20	15.6 mm	9.9 mm	21/5	11/13/2	175 mm^2^	3.3 mm
	0.0225	0.3176	0.0146	0.006	0.1670	0.1126	0.1358	0.1638	0.0439	0.2797
IOM	22.7 mm	29.9 mm	33.2 mm	7/12	15.3 mm	9.6 mm	15/4	6/10/3	181 mm^2^	3.3 mm
	22.4 mm	28.6 mm	32.7 mm	6/9	17.5 mm	9.2 mm	10/5	7/6/2	203 mm^2^	4.1 mm
	0.8868	0.3050	0.6266	0.8508	0.1826	0.6561	0.4203	0.6644	0.4804	0.4323

**Table 4:** Results regarding infraorbital paresis before treatment (BT), after any kind of treatment (AT), and improvement of infraorbital paresis after treatment (IAT). The results for the symptom present are presented above the results for the symptom absent. The results for PVIII are presented as YES/NO and for PV as A/B/C. The *p*-values for the appropriate statistical tests are presented below.

	PI	PII	PIII	PV	PVI	PVII	PVIII	PX	PXI
BT	21.9 mm	29.0 mm	32.6 mm	1/1/6	15.28 mm	9.6 mm	5/3	179 mm^2^	3.6 mm
	22.4 mm	29.5 mm	33.1 mm	5/11/15	16.5 mm	9.4 mm	21/10	194 mm^2^	3.6 mm
	0.8416	0.7307	0.6816	0.3711	0.5254	0.8620	0.7792	0.6602	0.9880
AT	21.4 mm	29.4 mm	33.7 mm	0/1/3	14.6 mm	10.7 mm	4/0	185 mm^2^	4.3 mm
	22.7 mm	29.5 mm	32.8 mm	4/9/13	16.4 mm	9.8 mm	16/10	192 mm^2^	3.3 mm
	0.6567	0.9527	0.6007	0.5718	0.4516	0.5598	0.1287	0.8825	0.5674
IAT	23.4 mm	29.8 mm	32.5 mm	1/1/1	14.9 mm	8.3 mm	2/1	183 mm^2^	2.7 mm
	22.6 mm	29.5 mm	33.1 mm	3/9/14	16.5 mm	10.1 mm	17/9	195 mm^2^	3.6 mm
	0.8157	0.8884	0.7500	0.5637	0.5742	0.0031	0.9647	0.9017	0.6134

## Data Availability

The detailed, anonymous data used in this study can be obtained from the first author upon reasonable request.

## Abbreviations

The following abbreviations are used in this manuscript:

AO	Arbeitsgemeinschaft für Osteosynthesefragen
CT	Computed tomography
BT	Before treatment
AT	After treatment
IAT	Improvement after treatment
AEP	Asymmetric eyeball placement
IOM	Impaired ocular mobility
PI	Parameter I
PII	Parameter II
PIII	Parameter III
PIV	Parameter IV
PV	Parameter V
PVI	Parameter VI
PVII	Parameter VII
PVIII	Parameter VIII
PIX	Parameter IX
PX	Parameter X
PXI	Parameter XI

## Appendix A

The follow-up periods were of different lengths, depending mostly on the treatment results, as patients not reporting symptoms after treatment often had a shorter follow-up period. In contrast, patients reporting symptoms after treatment often had a longer follow-up, sometimes ending with a second operation. In case multiple follow-up examinations were available in the documentation, a symptom was regarded as present after treatment for the purpose of this study if it was reported in the last follow-up examination for which data were available, or the last follow-up examination prior to a second surgery. The average follow-up time was 43 days, with a median of 15 days.

## Appendix B

The Statistica computer program was used to perform the statistical evaluation (v8.0, TIBCO Software, Palo Alto, CA, USA). The first stage of the evaluation was performed by means of descriptive statistics. For continuous variables, the distribution was tested by means of the Shapiro–Wilk test (as the sample sizes were below 50). Besides the age of the patients, all other variables were found to follow normal distribution. Since the study was performed in the hope of finding potential prognostic factors, all the parameters, I–XI, were tested for correlation with the symptoms. Moreover, tests regarding the patients’ sex, age, hospitalization time, time to last control, and side of the fractures, all tested against both the parameters and the symptoms, were performed. For combinations of discrete and continuous variables, a parametric test was performed (the only exception being combinations involving the age). The ratio of variances was tested by means of the F-test. If the hypothesis was not found to be true, the Cochrane–Cox test was employed; otherwise, Student’s *t*-test was used. For the calculations regarding age, the nonparametric Mann–Whitney U test was selected, being the best nonparametric alternative to Student’s *t*-test (with the exception of combinations with parameters V and IX, which had more than two possible results, and therefore were tested with the Kruskall–Wallis test. All combinations of two discrete variables were tested by means of the Chi-squared test, and if the result was found to be significant, additionally by means of Fisher’s exact test for comparison. If not stated otherwise, the significant results presented in the article are the *p*-values from Fisher’s exact test. In cases of two continuous variables, the Pearson correlation efficient and Spearman’s rank correlation coefficient were performed. The significance was tested by means of Student’s *t*-test.

## Appendix C

As stated in the section “Results”, data regarding some of the symptoms at admission could sometimes not be gathered. Possible reasons included massive swelling of the injured tissue combined with a periorbital hematoma, making a proper examination in terms of diplopia, ocular mobility, and asymmetric eyeball placement difficult, as well as alcoholic intoxication at admission, which rendered the patients’ complaints of diplopia doubtful. In some of those cases, no reliable pretreatment data from a secondary examination after admission were available, e.g., when surgical treatment was performed quickly. In these situations, the symptom was regarded as neither present nor absent before treatment, and CT-based data regarding the fracture were not used in the statistical evaluation regarding this specific symptom. Please note that the same CT-based data were still used in statistical tests regarding other symptoms for which data were available.

## Appendix D

For the purpose of this study, patients with a symptom before treatment but without it at last control were regarded as patients with improvement. On the other hand, patients with a symptom before and after treatment, as well as patients without the symptom before and after treatment and patients without the symptom before but with the symptom after treatment, were regarded as patients without improvement.
